# The aberrant expression of microRNAs and correlations with T cell subsets in patients with immune thrombocytopenia

**DOI:** 10.18632/oncotarget.12949

**Published:** 2016-10-27

**Authors:** Lu Liu, Mingqiang Hua, Chuanfang Liu, Na He, Zhao Li, Daoxin Ma

**Affiliations:** ^1^ Department of Hematology, Qilu Hospital, Shandong University, Jinan, China

**Keywords:** immune thrombocytopenia, microRNA, T helper cells, corticosteroid, antiplatelet antibody, Immunology and Microbiology Section, Immune response, Immunity

## Abstract

Both microRNAs and T helper (Th) cells involve in autoimmune diseases and their effects and interactions in immune thrombocytopenia (ITP) remain unclear. In the present study, we investigated the expression profiles of seven immune-related microRNAs (miR-155, 146a, 326, 142-3p, 17-5p, 21 and 181a) and the frequencies of four Th cells (Th1, Th2, Th17 and Treg) in peripheral blood mononuclear cell (PBMCs) of ITP patients and healthy controls. Platelet autoantibodies specific for GPIIb/IIIa or GPIb/IX were measured using MAIPA method. The regulating effect of miR-146a on Th differentiation was evaluated after using agomir. Our results showed that the expression of miR-146a, miR-326 or miR-142-3p in ITP patients was lower than that of controls. The frequencies of Treg cells were decreased, whereas the frequencies of Th17 and Th22 cells were increased significantly in ITP patients compared to those in controls. The expression levels of miR-142-3p and miR-146a were negatively correlated with Th17 cells, respectively. The expression of miR-146a was positively correlated with the frequencies of Treg cells and platelet counts. No significant correlation was found between the miRNAs expression and different autoantibody groups. The up-regulated miR-146a expression with agomir contributed to the differentiation of Th17 and Treg in ITP patients. Moreover, miR-146a was increased in the presence of DEX in PBMCs of ITP patients in vitro. Our study represents the abnormal expression profile of immune-related miRNAs in ITP patients, and miR-146a may be involved in Tregs differentiation and function.

## INTRODUCTION

Immune thrombocytopenia (ITP) is an acquired autoimmune disease characterized by accelerated platelet destruction and decreased peripheral platelet count [1]. Autoantibodies against platelet that result in platelet over-destruction are considered to be correlated with the pathophysiology of ITP. Moreover, T cells-mediated immune disturbance and cytotoxicity have been thought to be involved in its pathogenesis and disease progression [2-6]. However, the cellular immune abnormalities and their mechanism in ITP patients remain unclear.

T helper (Th) cells involve in the development and progression of inflammatory and autoimmune diseases and tumors. Th17 and Th22 cells are two novel Th subsets important for peripheral immune responses. Th17 is a unique CD4+ Th subset characterized by production of interleukin-17 (IL-17). Th17 may have evolved for host protection against microbes that Th1 or Th2 immunity is not well suited for. Recent data in humans and mice suggest that Th17 cells involve in the pathogenesis of a diverse group of immune-mediated diseases as well as tumors. Recently, a novel subset of CD4+ Th cells, IL-22-producing Th cells (Th22), have been identified and showed to challenge the classical Th1/Th2 paradigm [7, 8]. Th22 cells are inflammatory CD4+ T cells that secrete IL-22 but do not express IL-17 or interferon-gamma (IFN-γ). Th22 cells have been shown to be important in the pathogenesis of many autoimmunity diseases. The discovery of Th17 and Th22 cells has opened up a new avenue for research into the etiology and treatment of a broad spectrum of diseases. Emerging evidence suggests that the imbalance of Th subsets contributes to the development of immune-related diseases, such as myelodysplastic syndrome (MDS) [9], ankylosing spondylitis (AS) and rheumatoid arthritis (RA) [10]. We and others have previously demonstrated that Th1, Th17 and Th22 cells were elevated, while the frequencies and/or function of Treg cells were suppressed in the peripheral blood of ITP patients[7, 8, 11], indicating a vital role of Th imbalance in the pathogenesis of ITP. However, the molecular mechanisms that underline Th imbalances in ITP remain unknown.

MicroRNAs(miRNAs) are a novel class of short (21-25 nucleotides), non-coding, evolutionary conserved RNAs that widely exist in plant, animal and other eukaryotes. MiRNAs regulate post-transcriptional gene expression through incomplete base pairing with the 3′-untranslated region (3′-UTR) of target mRNAs to degrade the target mRNAs or inhibit translation[12-14]. Recently, some studies indicated that miRNAs may involve in innate immunity and adaptive immunity by regulating immune cell differentiation and signal transduction[12]. It was demonstrated that miR-181a is a key molecule in modulating signal strength of T cell receptor (TCR) [15]. The expression of miR-150 is increased during the process of T cell maturation, while decreased rapidly in Th1 and Th2 cells [16]. Our and other studies found that miRNA were aberrantly expressed in ITP patients [17, 18], indicating they could be involved in the pathogenesis of ITP. Therefore, the imbalance of Th subsets may be related to the aberrant expression of miRNAs in ITP. However, to date, there is no data about the roles and relationship between miRNAs and Th cells in ITP patients.

In this study, we measured the expression of seven immune-related miRNAs (miR-155, miR-146a, miR-326, miR-142-3p, miR-17-5p, miR-21and miR-181a) and the frequencies of Th subsets (Th1, Th17, Th22 and Tregs) to investigate their profile and relationship in the pathogenesis of ITP. Moreover, their clinical relevance including response to treatment was also evaluated.

## RESULTS

### MiRNAs were aberrantly expressed in ITP patients

**Figure 1:**
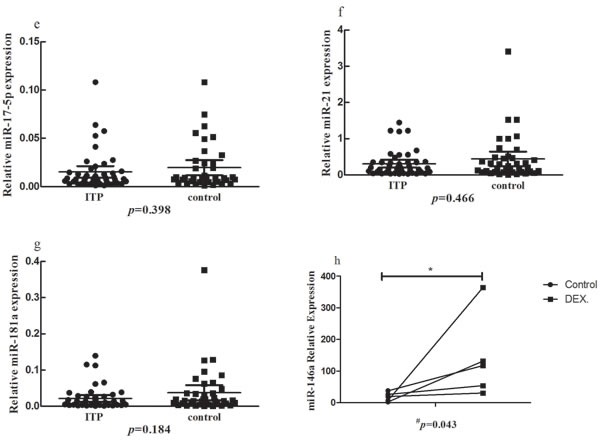
The expression of seven microRNAs in ITP patients and controls **b.**, **c.**, **d.** The expression of miR-146a, miR-326 or miR-142-3p in the PBMCs of ITP patients was decreased significantly compared to that in healthy controls (*P* < 0.05). **a.**, **e.**, **f.**, **g.** No significant change was found for miR-155, miR-17-5p, miR-21 or miR-181a. **h.** The expression of miR-146a was increased in the presence of dexamethasone.

We studied the expression of seven immune-related miRNAs (miR-155, miR-146a, miR-326, miR-142-3p, miR-17-5p, miR-21 and miR-181a) in PBMCs of ITP patients and healthy controls. The expression of miR-146a, miR-326 or miR-142-3p in ITP patients was decreased significantly compared to that in healthy controls (*P* < 0.05). Though miR-155 was elevated in ITP patients, it was not reach significant difference (P = 0.117). As for miR-17-5p, miR-21 and miR-181a, no statistical significance was found (Figure 1a-1g and Table 1). We also analyzed the expression of seven miRNAs in newly-diagnosed and recurrent ITP patients, and no statistical significance was found.

**Table 1:** Relative expression of miRNA in the two groups

miRNA	ITP (*n* = 46)		Healthy controls (*n* = 39)	
	median	range	median	median
miR-155	2.93×10^−2^	(0.53-11.74)×10^−2^	2.37×10^−2^	(0.47-9.09)×10^−2^
miR-146a	7.27×10^−2^	(0.75-18.30)×10^−2^	8.84×10^−2^	(3.54-69.74)×10^−2^
miR-326	2.51×10^−4^	(0.24-37.99)×10^−4^	4.37×10^−4^	(0.31-139.85)×10^−4^
miR-142-3p	0.71	0.02-2.77	0.89	0.19-5.46
miR-17-5p	0.86×10^−2^	(0.12-11.03)×10^−2^	0.90×10^−2^	(0.13-10.81)×10^−2^
miR-21	0.19	0.03-1.44	0.20	0.01-3.41
miR-181a	0.93×10^−2^	(0.073-13.97)×10^−2^	1.51×10^−2^	(0.09-37.63)×10^−2^

Data were shown as median and range

### Positive correlations were found among the seven miRNAs in ITP patients

**Figure 2:**
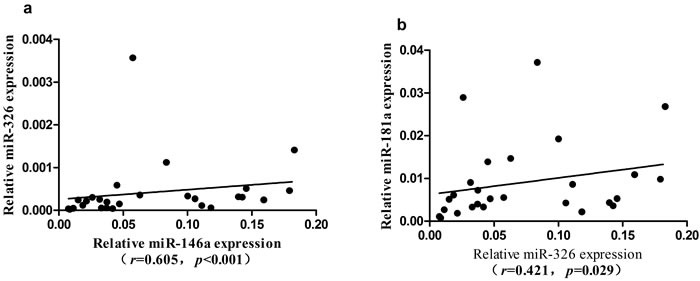
Correlation of the expression among seven microRNAs in ITP group **a.** The expression level of miR-326 was positively correlated with miR-146a. **b.** The expression level of miR-326 was positively correlated with miR-181a.

In PBMCs of ITP patients, the expression of miR-326 showed a significantly positive correlation with the expression of miR-146a or miR-181a (*r* = 0.605, *P* < 0.001 or *r* = 0.421, *P* = 0.029, respectively) (Figure 2). No significant correlation was found between other miRNAs.

### Clinical relevance of miRNAs in ITP patients

**Figure 3:**
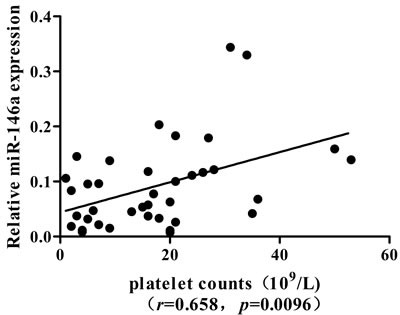
Correlation of miR-146a expression with peripheral platelet count in ITP patients

We next analyzed the association between miRNAs and platelet counts in ITP patients. A significantly positive correlation was found between the expression of miR-146a and platelet counts (*r* = 0.658, *P* = 0.0096) in ITP patients (Figure 3). No significant difference was found between the expression of miRNAs and clinical characteristics, including age and gender.

Thirty-one hospitalized patients received systematically corticosteroid therapy. Twenty-three patients were in response (R) while the other eight patients were in no response (NR), and the response rate for therapy was up to 74.19%. The expression level of seven miRNAs was of no significant difference between the R group and NR group (*P* > 0.05, data not shown).

### MiR-146a was increased in the presence of dexamethasone (DEX) in PBMCs of ITP patients *in vitro*

To determine the influence of DEX on the expression of miR-146a, we measured the change of miR-146a from PBMCs of ITP patients in the presence of DEX *in vitro*. As shown in Figure 1h, the expression of miR-146a was increased after treatment with 0.5μM DEX compared with negative control (*P* = 0.043).

### The frequencies of Th17, Th22 and Treg cells were imbalanced in ITP patients

**Figure 4:**
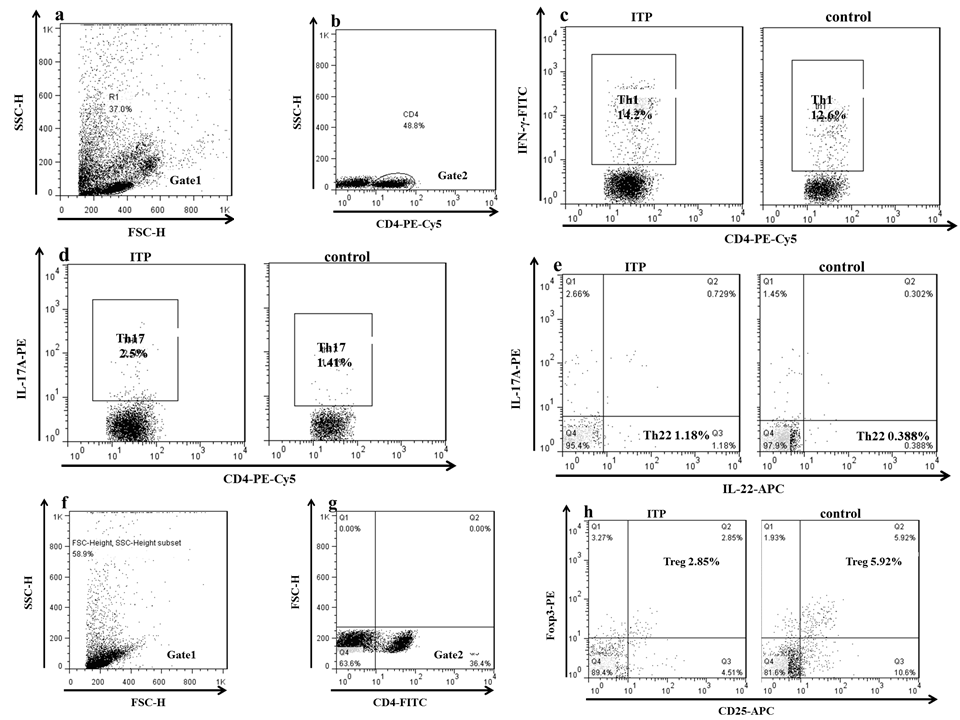
The representative plots of Th1, Th17 Th22 and Treg cells in ITP patients and controls **c.** The percentage of Th1 cells. **d.** The percentage of Th17 cells. **e.** The percentage of Th22 cells.**g.** CD4+ T lymphocytes.**h.** The percentage of Treg cells.

**Figure 5:**
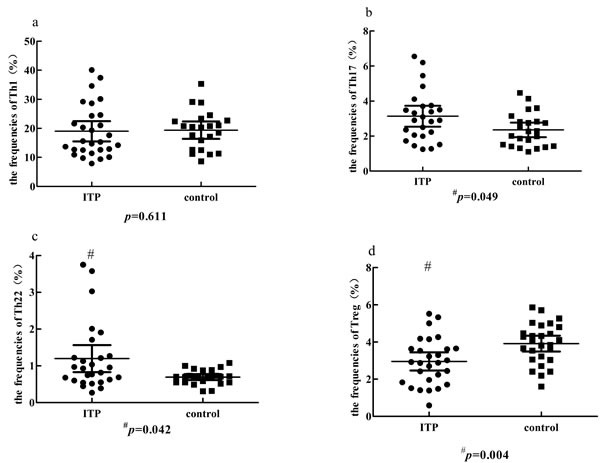
Comparison of the percentage of Th1, Th17, Th22 and Treg cells in ITP group and control group **a.** No significant change of Th1 cells was found between ITP patients and controls. **b.**, **c.** The percentage of Th17 or Th22 cells was significantly higher in ITP than in controls (^#^*p* < 0.05). **d.** A significantly lower Treg percentage was observed in ITP patients (^#^*P* < 0.05).

The typical dot plots of Th cells in representative ITP patients and healthy controls were shown in Figure 4. Compared with healthy controls (median, 4.06%; range, 1.6-5.86%), the frequencies of Treg cells (median, 2.9%; range, 0.59-5.52%; *P* = 0.004) were decreased in ITP patients (Figure 5d). Whereas, the frequencies of Th17 (median, 2.9%; range, 1.25-6.55%; *P* = 0.049) and Th22 cells (median, 0.93%; range, 0.27-3.75%; *P* = 0.042) in ITP patients were increased significantly compared to those in healthy controls (Figure 5b, 5c). As for Th1 cells, no statistical change was found (Figure 5a). We also analyzed the frequencies of Th subsets in newly-diagnosed and recurrent ITP patients, and no statistical significance was found.

### MiRNAs were correlated with Th subsets in ITP patients

**Figure 6:**
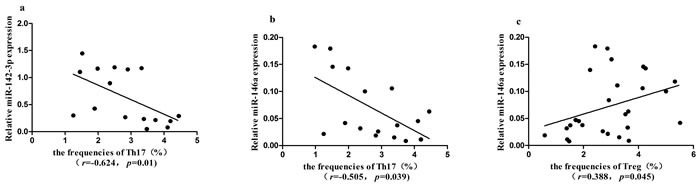
Correlation between Th1, Th17, Th22, Treg cells and the expression of seven miRNAs in ITP patients **a.**, **b.** The expression level of miR-142-3p or miR-146a was negatively correlated with Th17 cells, respectively (*P* < 0.05). **c.** The expression level of miR-146a was positively correlated with Treg cells (*P* < 0.05).

To analyze whether the miRNAs correlated with T cell subsets in ITP patients, we performed Spearman's test. The expression levels of miR-142-3p (*r* = −0.624, *P* = 0.01, Fig.6a) and miR-146a (*r* = −0.505, *P* = 0.039, Figure 6b) were negatively correlated with Th17 cells, respectively. The expression of miR-146a was positively correlated with the frequencies of Treg cells (*r* = 0.388, *P* = 0.045) in ITP patients (Figure 6c). The expression of miR-142-3p was marginally inversely correlated with the frequencies of Th22 cells (*r* = −0.371, *P* = 0.057).

### The profile of miRNAs or Th subsets between autoantibody-positive and autoantibody-negative ITP patients

Twenty-six ITP patients were detected the antiplatelet autoantibodies, including 13 patients for positive autoantibodies, 5 patients for only one positive autoantibody and 8 patients for negative autoantibodies. The expression levels of seven miRNAs and the frequencies of T cell subsets were of no significant difference between the different autoantibody groups (*P* > 0.05, data not shown).

### Alteration of miR-146a expression contributed to the differentiation of Th17 and Treg in ITP patients

**Figure 7:**
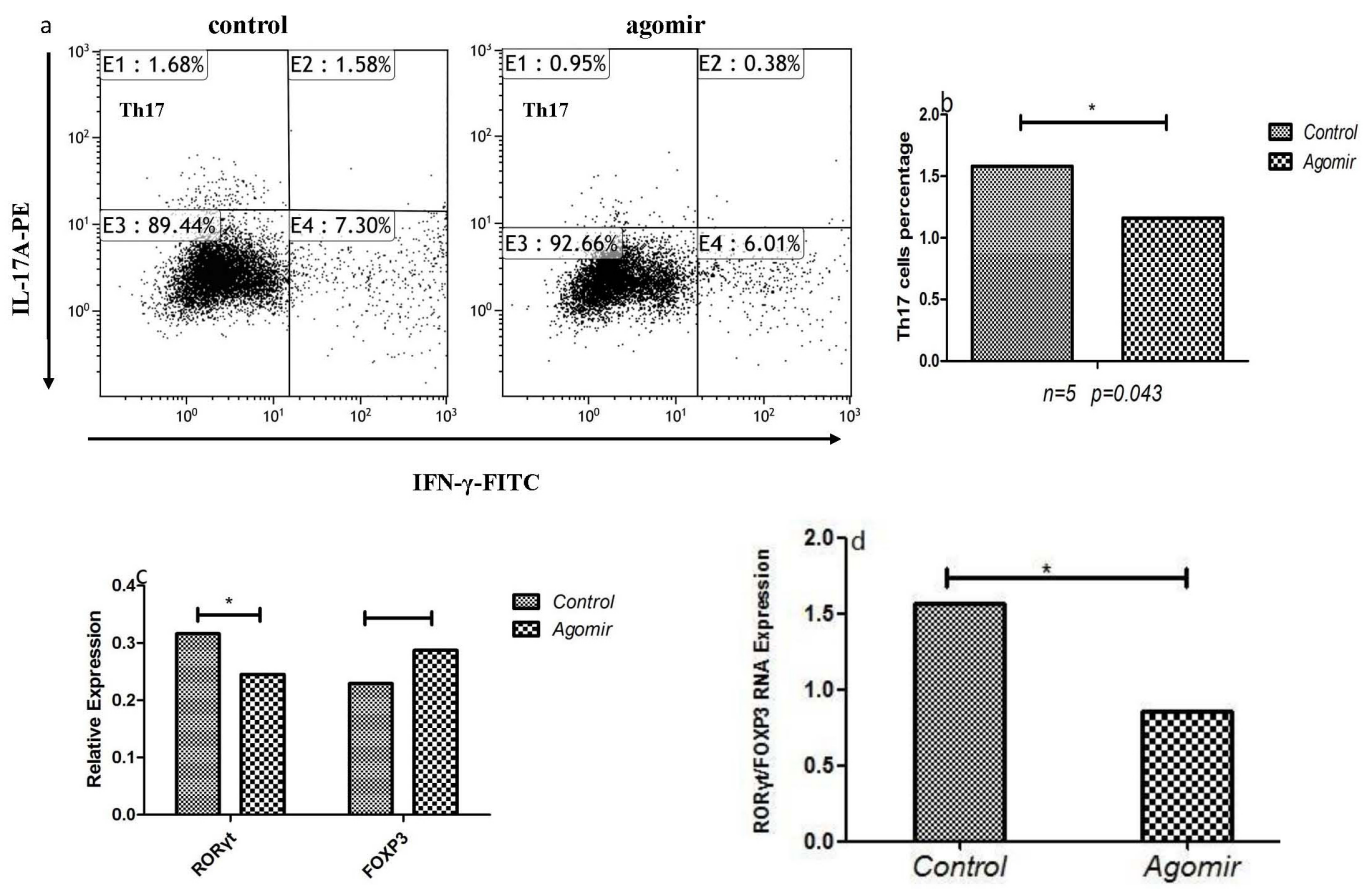
The effects of miR-146a on Th cells of ITP patients **a.**, **b.** The frequency of Th17 was decreased after up-regulating miR-146a with agomir. **c.**, **d.** The expression of FOXP3 was increased while the RORγt expression and the ratio of RORγt/FOXP3 were decreased after up-regulating miR-146a with agomir.

To determine the effect of miR-146a on Th cells differentiation, PBMCs of ITP patients were cultured with or without miR-146a agomir for 4 days in condition of stimulating factors of T lymphocytes. Agomir is a small double-stranded RNA, especially chemical modification by simulating endogenous miRNA to adjust the biological function of the target genes and dedicates to inhibit endogenous miRNA efficient inhibitors. The results showed that miR-146a expression in ITP patients was significantly increased after being transfected with miR-146a agomir. As shown in Figure 7a and 7b, the percentage of Th17 cells from ITP patients were decreased after up-regulating the expression of miR-146a (*p* = 0.043). Moreover, Tregs have the increased trend after miR-146a agomir transfection in PBMCs of two ITP patients.

We also detected the Th associated transcriptional factors. In accordance with Th cell differentiation, a significant decrease of RORγt mRNA and an increased trend for FOXP3 mRNA were found in PBMCs that transfected with miR-146aagomir. The ratio of RORγt /FOXP3 was also analyzed, and it was shown reduced after up-regulation of miR-146a expression (Figure 7c and 7d).

## DISCUSSION

Recent studies have shown that miRNAs are essential for normal immune T cell function, and implicated in the pathogenesis of autoimmune diseases [19-21]. However, the profile and relationship of miRNAs and Th cells in ITP remain unclear.

Here, we showed that miR-146a, a miRNA previously reported aberrant expression in many autoimmune diseases, was decreased in ITP patients. We also demonstrated that increased Th17 and decreased Treg cells in ITP patients, and miR-146a showed higher correlation with peripheral platelet counts and the frequencies of Th17 and Tregs. Our result showd that miR-146a was increased in the presence of DEX in PBMCs of ITP patients *in vitro*. In our previously demonstrated that the percentage of Tc17 and Th17 cells decreased with the increase in the concentration of DEX[8]. It was proven that miR-146a was highly expressed in Treg cells, and the latter can inhibit the differentiation of Th17 cells. Inhibition of RORγt activity or induction of Foxp3 restrains the differentiation of inflammatory Th17 cells in response to TGF-β[22]. Rudensky et al have demonstrated that miR-146a was critical for the suppression function of Treg cells, and contributed to Treg-mediated control of Th1 responses in mice[34]. These findings suggest that miR-146a may be involved in the pathophysiologic procedures of ITP. Joanna *et al*. demonstrated miR-146a was remarkably up-regulated in mouse and human megakaryopoiesis. They also found that overexpression of miR-146a in lympho-myeloid cells does not inhibit megakaryopoiesis indirectly by suppressing innate immunity, in contrast to the megakaryocyte-enhancing effects of miR-146a haploinsufficiency [23]. It is possible that overexpressed miR-146a could suppress megakaryocytosis caused by inflammatory stress. In our result, miR-146a expression was decreased. A significant positive correlation was found between miR-146a expression and platelet counts in ITP patients, suggesting the important role of miR-146a in ITP. Further studies in knockout and transgenic animal models would better identify the role of miR-146a in ITP.

We also found the expression of miR-326 was decreased in PBMCs of ITP patients. In recent years, studies have predicted several possible target genes of miR-326 such as PKM2, VDR and Ets-1. Ets-1, a validated target gene of miR-326, involved in the differentiation of Th17 cells in multiple sclerosis (MS) and experimental autoimmune encephalomyelitis (EAE) [24]. On the other hand, the upregulation of Ets-1 can enhance the promoter activity of miR-146a and regulate the expression of miR-146a directly [25]. In our studies, we confirmed there was a shift of Th17 in ITP patients. We found the expression of miR-326, highly correlating with the expression of miR-146a, was decreased in ITP patients. Therefore, we speculate that miR-326 and miR-146a may contribute to the progression of ITP with Ets-1 through a same mechanism. More studies are necessary to clarify the specific role of miR-326 in ITP patients.

MiR-142-3p has been shown mainly involved in tumor-associated pathway, and its immunoregulatory effect has attracted more and more concern in recent years. In our study, the expression of miR-142-3p was decreased in PBMCs in ITP patients. It has confirmed that the specifically target gene of miR-142-3p was CD84, which belongs to the signaling lymphocytic activation molecule (SLAM) family. Furthermore, inhibiting miR-142-3p expression in healthy CD4^+^ T cell caused T cell overactivation and B cell hyperstimulation, which produce more IgG antibody to induce immune response [26]. We found the expression of miR-142-3p was marginally inversely correlated with the frequencies of Th22 cells. Therefore, we speculate that miR-142-3p may also be involved in activation of self-reactive T cells in ITP patients. Detailed mechanisms of miR-142-3p in ITP will be clarified in our future work.

We sought to investigate the expression profiles of seven immune-related miRNAs in ITP patients. As for miR-155, miR-17-5p, miR-21 and miR-181a, no statistical significance was found. MiR-155 is increased in activated immune cells and involves in immune responses by regulating cell differentiation, antibody production, and cytokine secretion [27, 28]. MiR-21 has been widely implicated in the regulation of inflammatory disorders, and it was found increased in psoriasis, atopic eczema, and SLE [29, 30]. Although the expression of miR-181a was no significant differences between ITP patients and healthy control in our result, it show significant positive correlation with the expression of miR-146a. Other study demonstrated that higher miR-181a expression correlates with greater T cells sensitivity in immature T cells, and it can represses multiple negative regulators in the TCR signaling pathway [31]. We speculate miR-181a may correlate with the progression of ITP.

This study represents the expression profile of seven immune-related miRNAs in ITP patients, and miR-146a may be involved in Tregs differentiation and function. The abnormal expression of miRNAs may participate in the occurrence and development of ITP, which indicated that they could potentially serve as the novel directions for diagnosis and treatment of ITP.

## MATERIALS AND METHODS

### Patients and controls

A total of 46 newly-diagnosed and recurrent ITP patients (19 males and 27 females; median age, 30 years; age range, 16-65 years) were enrolled in this study. Enrollment occurred in the department of Hematology of Qilu Hospital, Jinan, China from May 2008 to June 2013. The platelet counts range from 2 to 56×10^9^/L, with a median count of 16×10^9^/L. All patients met the diagnosis criteria of ITP as described before [1]. Exclusion criteria are patients who had diabetes, pregnancy, active or chronic infections, or connective tissue diseases. Thirty nine healthy controls were composed of 17 males and 22 females with a median age of 27 years (range 24-50 years). There was no significant difference of age or gender between ITP patients and healthy controls. Our study was approved by the Medical Ethical Committee of Qilu Hospital of Shandong University. Informed consent was obtained for each subject before being included in the study.

### Treatment regimen and response criteria

High-dose dexamethasone was 40mg/d/po for four days, or prednisolone was 0.5-1mg/kg.d/po. After platelet counts increased, the dose of drug was gradually reduced (< 15mg/d). If no response in four weeks, dexamethasone or prednisolone should be sharply deceased and discontinued.

The response criteria of ITP are as follow[32]:Complete response (CR): platelet count ≥100× 10^9^/L and absence of bleeding; Response (R): platelet count ≥30×10^9^/L and at least 2-fold increase the baseline count, absence of bleeding; No response (NR): platelet count < 30×10^9^/L or less than 2-fold increase of baseline or the presence of bleeding. Platelet count must be measured on 2 occasions > 7 days.

### Sample preparation

PBMCs were isolated by Ficoll-Hypaque density gradient centrifugation and stored at -80°C. PBMCs were used for functional study and quantitative reverse transcription polymerase chain reaction (RT-PCR). Heparinized peripheral whole blood was used for flow cytometric analysis.

### PBMCs transfection and culture

PBMCs from ITP patients were transfected with 20nmol/LmiR-146a agomir along with negative control(Shanghai Genepharma, China), using EndoFectin-Max Kits (GeneCopoeia, USA) together with OPTI-MEM Reduced Serum Medium (Gibco, USA) according to the manufacturer's instructions. Then, cells were stimulated with 2μg/mL anti-CD3 antibody and 2μg/mL anti-CD28 antibody in the presence of 50 U/mL IL-2 and cultured in RPMI-1640 containing 10% fetal bovine serum (FBS) (Gibco, Australia). Quantitative RT-PCR was applied to determine the up-regulated expression of miR-146a using All-in-One miRNA qRT-PCR Detection Kit (GeneCopoeia, USA) according to the manufacturer's instructions. Four days later, cells were collected for further analysis of Th cells differentiation (flow cytometry, Quantitative RT-PCR).

### Addition of DEX to PBMCs from patients with ITP

PBMCs were adjusted to 1×10^6^/mL in RPMI-1640 culture medium supplemented with 10% FBS at a density of 1×10^6^/well in a 24-well plate. The cells were incubated in humidified air in 5% CO_2_ at 37°C in the presence of 0 or 0.5 μmol/L of DEX. After 72 h, the PBMCs were collected to determine the expression of miR-146a.

### Quantitative RT-PCR analysis of miRNA and Th cells transcriptional factors

Total RNA was extracted from PBMCs or cultured cells using TRIzol (Invitrogen) according to the manufacturer's protocol. RNA concentration and purification were measured by a spectrophotometer (Eppendorf, GER).

For the detection of miRNAs, RT was performed according to the manufacturer's instruction of TaqMan^®^ MicroRNA Reverse Transcription Kit (Applied Biosystems). The reaction system was incubated at 16 °C for 30 min, 42 °C for 30 min and 95 °C for 5 min. Quantitative PCR was performed using the TaqMan^®^ Universal Master Mix (Applied Biosystems) on a Roche LightCycler 480II instrument. The reaction system was incubated at 50 °C for 5 min and 95 °C for 10 min, and followed by 40 cycles of denaturation at 95 °C for 15 s and extension at 60 °C for 1 min. The primers and probes used for RT and qRT-PCR were provided from TaqMan^®^ MiRNA Assays. U6 snRNA was used as endogenous control. All samples were analyzed in triplicates. Results were analyzed as 2^−ΔCt^.

For the detection of Th transcriptional factors, cDNA was synthesized with Prime Script RT Master Mix Kit (Takara Bio Inc., China). Reverse transcription reaction was done at 37 °C for 15 min, followed by 85 °C for 5 s. The real-time PCR contained, in a final volume of 10 μL, 5 μL of 2× SYBR Green Real-time PCR Master Mix (TOYOBO, Japan), 1 μL of cDNA, 3.2μL of ddH_2_O and 0.4μL of the forward and reverse primers, respectively. The primers were shown as follows: RORγt Forward 5′-CAA TGG AAG TGG TGC TGG TTA G -3′, Reverse 5′-GGG AGT GGG AGA AGT CAA AGA T-3′; FOXP3 Forward 5′- GGA AAG GAG GAT GGA CGA ACA -3′, Reverse 5′- GGA AAC CTC ACT TCT TGG TCC C -3′; GAPDH Forward 5′-GCT CTC TGC TCC TCC TGT TC-3′, Reverse 5′-GTT GAC TCC GAC CTT CAC CT -3′. All experiments were conducted in triplicate. The PCR products were analyzed by melt curve analysis and agarose gel electrophoresis to determine product size and to confirm that no by-products were formed. The results were expressed relative to the number of GAPDH transcripts using the comparative Ct method formula 2 ^−ΔCt^.

### Flow cytometric analysis of Th22, Th17 and Th1 cells

The heparinized peripheral blood (200μl) or cultured cells were incubated for 4h at 37°C in 5% CO_2_ with an equal volume of Roswell Park Memorial Institute (RPMI)-1640 medium, 25ng/ml of phorbol myristate acetate (PMA), 1μg/ml of ionomycin, and 1.7μg/ml of monensin (all from Alexis Biochemicals, San Diego, CA, USA). Then cells were stained with PE-Cy5-conjugated anti-CD4 monoclonal antibodies (MAbs) at room temperature for 20 min. After being fixed and permeabilized, cells were stained with FITC-conjugated anti-IFN-γ MAbs, PE-conjugated anti-IL-17A MAbs and APC-conjugated anti-IL22 MAbs at room temperature for 20min. Isotype controls were given to correct compensation and confirm antibody specificity. Stained cells were analyzed using a FACS Calibur cytometer equipped with CellQuest software (BD Bioscience PharMingen, San Jose, CA, USA). All the antibodies were purchased from eBioscience, San Diego, CA, USA. For analysis, we first gated CD4+ lymphocytes, then analyzed the proportion of Th1 (CD4^+^IFN-γ^+^), Th17 (CD4^+^ IL17^+^) and Th22 (CD4^+^IFN-γ^−^IL17^−^IL-22^+^) in CD4+ lymphocytes.

### Flow cytometric analysis of Treg cells

PBMCs (2×10^6^) isolated from the remaining blood were stained with FITC-conjugated anti-CD4 MAbs, APC-conjugated anti-CD25 MAbs at 4°C for 30min. After washing, the cells were fixed and permeabilized according to the manufacturer's instruction of Foxp3 staining Buffer set (eBioscience, USA). For intracellular cytokines staining, cells were stained with PE-conjugated anti-Foxp3 MAbs at 4°C for 30min. The percentages of CD4+CD25+Foxp3+ Treg cells were determined for all participants.

### The modified MAIPA assay

All plasma samples were stored at -80°C until anti-platelet autoantibody determination. The presence of specific antibodies to anti-platelet GPIIb/IIIa and/or GPIb/IX was analyzed by modified monoclonal antibody-specific immobilization of platelet antigens (MAIPA). The assay was carried out as previously described in detail by Hou et al [33].

### Statistical analysis

Results were expressed as median and range. Comparisons between two groups were assessed by the rank sum test, and Spearman's test was used for correlation analysis. Differences among multiple groups, the data of which were not normally distributed, were assessed by Kruskal-Wallis test (H test). All tests were performed by SPSS 19.0 system. *P* value < 0.05 was considered statistically significant and *P* value < 0.01 considered highly statistically significant.
